# Impact of diuretics use over AKI development and hospital mortality in ICU patients

**DOI:** 10.1186/2197-425X-3-S1-A458

**Published:** 2015-10-01

**Authors:** E Banderas-Bravo, G Seller-Pérez, J Barrueco-Francioni, I De-Dios-Chacón, M Delgado-Amaya, D Arias-Verdú, M Herrera-Gutierrez

**Affiliations:** Complejo Universitario Carlos Haya, ICU, Malaga, Spain; Hospital Son-Llatzer, Palma de Mallorca, Spain

## Introduction

In different studies, use if diuretics have been shown not useful for AKI prevention and a possible relationship with mortality has been suggested.

## Objectives

We intend to analyze this issue by means of propensity score methodology (PS).

## Methods

Post-hoc analysis of a prospective observational cohort from a previous study on AKI conducted in our Unit. We defined AKI based in KDIGO II/III stages criteria. We calculated a PS for patients with or without diuretics by regression analysis and pairing 1:1 by quintiles grouping. Then performed an analysis for diuretic-AKI and diuretic-mortality by logistic regression, including the PS in it. Software R 3.1.2 para OsX. The Ethics Committee from our centre approved this work.

## Results

from 279 patients studied, 80 out of 142 patients with diuretic (56.3%) developed AKI against 56 out of 137(41.2%) without it (p < 0.01), OR 1.86 (IC 1.16-3.01). We computed a PS adjusting by age, gender, history of high blood pressure, diagnosis, basal creatinine and vasopressors. After the pairing an optimal balance was not reached for gender, diagnosis and base creatinine and these variables were included beside PS in the final logistic regression analysis. In this regression we did not detect any significant effect of the diuretic on AKI development, OR 1.26 (IC 95% 0.73-2.18).

Among patients without diuretic, mortality was 47.4% against 61.4% when diuretic was used (p < 0.001), OR 5.07 (IC 95% 2.89-8.86), absolute mortality difference 37.9% (IC 25.8-50.1). We computed a PS adjusting by age, diagnosis, APACHE, basal creatinine, infection and vasopressors. After the pairing an optimal balance was not reached for APACHE, infection and base creatinine and these variables were included beside PS in the final logistic regression analysis. In the final analysis, diuretic use retained a significant relationship with mortality, OR 3.26 (IC 95% 1.78-5.97).

## Conclusions

In our cohort, once confounding variables were controlled by propensity score methodology, diuretic use did not relate to AKI development but showed an independent relationship with hospital mortality.

